# The use of artificial intelligence to optimize medication alerts generated by clinical decision support systems: a scoping review

**DOI:** 10.1093/jamia/ocae076

**Published:** 2024-04-19

**Authors:** Jetske Graafsma, Rachel M Murphy, Ewoudt M W van de Garde, Fatma Karapinar-Çarkit, Hieronymus J Derijks, Rien H L Hoge, Joanna E Klopotowska, Patricia M L A van den Bemt

**Affiliations:** Department of Clinical Pharmacy and Pharmacology, University Medical Center Groningen, University of Groningen, Groningen, 9713GZ, The Netherlands; Department of Medical Informatics Amsterdam UMC, University of Amsterdam, Amsterdam, 1000GG, The Netherlands; Amsterdam Public Health Institute, Digital Health and Quality of Care, Amsterdam, 1105AZ, The Netherlands; Department of Pharmacy, St Antonius Hospital, Utrecht, 3430AM, The Netherlands; Division of Pharmacoepidemiology and Clinical Pharmacology, Utrecht University, Utrecht, 3584CS, The Netherlands; Department of Clinical Pharmacy and Toxicology, Maastricht University Medical Center, Maastricht, 6229HX, The Netherlands; Department of Clinical Pharmacy, CARIM, Cardiovascular Research Institute Maastricht, Maastricht University, Maastricht, 6229ER, The Netherlands; Department of Pharmacy, Jeroen Bosch Hospital, Den Bosch, 5200ME, The Netherlands; Department of Pharmacy, Wilhelmina Hospital, Assen, 9401RK, The Netherlands; Department of Medical Informatics Amsterdam UMC, University of Amsterdam, Amsterdam, 1000GG, The Netherlands; Amsterdam Public Health Institute, Digital Health and Quality of Care, Amsterdam, 1105AZ, The Netherlands; Department of Clinical Pharmacy and Pharmacology, University Medical Center Groningen, University of Groningen, Groningen, 9713GZ, The Netherlands

**Keywords:** artificial intelligence, clinical decision support systems, medication safety, medication alerts

## Abstract

**Objective:**

Current Clinical Decision Support Systems (CDSSs) generate medication alerts that are of limited clinical value, causing alert fatigue. Artificial Intelligence (AI)-based methods may help in optimizing medication alerts. Therefore, we conducted a scoping review on the current state of the use of AI to optimize medication alerts in a hospital setting. Specifically, we aimed to identify the applied AI methods used together with their performance measures and main outcome measures.

**Materials and Methods:**

We searched Medline, Embase, and Cochrane Library database on May 25, 2023 for studies of any quantitative design, in which the use of AI-based methods was investigated to optimize medication alerts generated by CDSSs in a hospital setting. The screening process was supported by ASReview software.

**Results:**

Out of 5625 citations screened for eligibility, 10 studies were included. Three studies (30%) reported on both statistical performance and clinical outcomes. The most often reported performance measure was positive predictive value ranging from 9% to 100%. Regarding main outcome measures, alerts optimized using AI-based methods resulted in a decreased alert burden, increased identification of inappropriate or atypical prescriptions, and enabled prediction of user responses. In only 2 studies the AI-based alerts were implemented in hospital practice, and none of the studies conducted external validation.

**Discussion and Conclusion:**

AI-based methods can be used to optimize medication alerts in a hospital setting. However, reporting on models’ development and validation should be improved, and external validation and implementation in hospital practice should be encouraged.

## Background and significance

Preventing adverse drug events (ADEs) is an essential part of medication safety efforts worldwide.^1^ ADEs can lead to serious harm and even death and contribute to excess healthcare costs.^2–4^ A recent retrospective cohort study in 11 hospitals in the United States showed that ADEs, despite various medication safety efforts, are still the most frequent (39.0%) type of adverse events patients encounter during their hospital stay, and are often deemed preventable (26.8%).^5^ Among the most broadly deployed tools to prevent ADEs in hospitalized patients, are clinical decision support systems (CDSSs).^6^

CDSSs generate medication alerts when the content of the electronic hospital record (EHR) of a patient matches with pre-defined rules in a knowledge database upon which CDSSs operate. The alerts are primarily shown during the medication prescribing phase. The goal of these alerts is to support healthcare providers in checking dosages, drug-drug interactions, contra-indications, duplicate therapy, drug allergies, and intolerances, and by doing so reducing the ADE risk of hospitalized patients. However, current CDSSs generate a high alert volume containing medication alerts with no or limited clinical relevance, resulting in alert fatigue and override rates as high as 96%. This alert fatigue is concerning as it may result in missing clinically significant alerts, which compromises patient safety.^7–11^

This is mainly caused by the fact that the current medication alerts fail to account for the complexity of care and patient specific variables.^10^^,^^12^^,^^13^ In the past years, several attempts have been made to improve appropriateness and clinical value of medication alerts. A recent scoping review of Ledger et al identified 6 types of interventions for medication alerts in hospitals: alert inactivation, alert severity reclassification, information provision, use of contextual information, threshold adjustment, and encounter suppression.^14^ A study by Bakker et al showed the effect of alert inactivation and severity reclassification; only drug-drug interaction alerts assessed as clinically relevant in the intensive care setting were turned on, which resulted in 12% decrease in the number of high-risk combinations.^15^ Selecting the alerts to be inactivated by employing a multidisciplinary committee has shown to be an effective approach.^16^ A way to use contextual information to optimize alerts is by designing computerized decision tree rules, to context-dependently suppress irrelevant drug-drug interaction alerts, which has been shown to result in less alerts and a higher positive predictive value (PPV).^17^ However, given the high number of medication alerts and high override rates, room for improvement remains. Medication alerts should be more specific, for example by targeting only high risk contraindications, or by tailoring the alerts to specific medical specialties.^18^^,^^19^

Artificial Intelligence (AI) methods may contribute to further optimization of medication alerts generated by CDSSs, eg, by predicting physician responses, generating AI-based medication alerts, or by developing a triage system. In contrast to rigid and simple decision tree like logic upon which current CDSSs operate, AI-based methods can take into account large amounts of EHR data, recognize complex patterns, and provide individualized predictions.^20^ In medicine, the most often used AI-based methods are machine learning (ML), deep learning (DL), and natural language processing (NLP).^21^ ML and DL can be applied to structured data, whereas NLP can be used on unstructured data. In ML data analytical algorithms are developed to extract features from data, which can be used to cluster patients characteristics or predict the probability of disease outcomes. DL is an extension of ML, which can be described as networks with a large number of layers, consequently leading to the ability to explore more complex non-linear patterns in the data. In NLP, information is extracted from unstructured data, such as clinical notes from physicians, to supplement and enrich medical data.^22^

Use of these methods to create new or optimize existing medication alerts may help to reduce alert fatigue, for example by prioritizing the alerts based on appropriateness and usefulness, but also in preventing ADEs which are currently missed by the existing CDSSs.^12^ Several literature reviews have already been performed on AI and CDSS in specific domains such as in oncology, dentistry, or infectious disease.^23–25^ However, none of these reviews focused on medication alerts generated by CDSS in hospitalized patients.

Therefore, the aim of this scoping review was to provide a comprehensive overview of the current state of applying AI-based methods to optimize medication alerts generated by CDSS in a hospital setting. By synthesizing the available evidence, this review aims to inform on the potential AI offers for improvement of CDSS and to identify opportunities for future research.

## Methods

### Approach

The proposed scoping review was conducted in accordance with the Joanna Briggs Institute (JBI) methodology for scoping reviews^26^ and reported according to the Preferred Reporting Items for Systematic Reviews and Meta-Analyses extension for Scoping Reviews (PRISMA-ScR).^27^ The protocol was registered in the Open Science Framework.^28^

### Information sources and search strategy

A preliminary search of Medline and Embase databases was conducted on May 10, 2023 before conducting this scoping review and no systematic or scoping reviews were identified on the specific topic of this review. Furthermore, at that time, no registered research protocols were found in the Open Science Framework or PROSPERO on this specific topic.

The literature search was conducted in Medline, Embase, and Cochrane Library (reviews and trials) databases on May 25, 2023 using the definite search strategy. No filters were applied with regard to publication year and country. The search was limited to the English language. The search strategy consisted of 3 segments: 1 related to AI-based methods, 1 related to pharmacotherapy, and 1 related to CDSS. The exact search strategies for each database can be found in Supplement Appendix S1. Additionally, the references of relevant studies were screened to identify possible other relevant studies. Forward citation was performed in Web of Science and in Scopus to identify additional relevant studies, using the studies initially labeled relevant during the title/abstract screening phase. If the studies identified via these additional searches were not already identified via the primary search strategy, the search strategy was reviewed and adjusted if needed to be able to include all relevant studies.

### Eligibility criteria

This scoping review aimed to include all studies that explore the use of AI-based methods to optimize medication alerts generated by CDSS in a hospital setting. We included a wide range of AI-based methods including supervised and unsupervised ML, NLP, and DL methods. Quantitative and mixed-method studies of any observational or interventional design (including but not limited to cohort studies, randomized controlled trials, and controlled trials) were eligible for inclusion. Scoping and systematic reviews or meta-analyses that met the inclusion criteria were not included but their references lists were screened for relevant studies. Qualitative studies, case reports, abstracts of congresses, expert opinions, editorials, and narrative reviews were excluded.

This review focusses on optimization of medication alerts generated by CDSSs. Optimization of medication alerts by applying AI can be achieved in different ways, eg, by predicting physicians responses, generating AI-based medication alerts, or developing a triage system. Regarding the type of medication alerts included, we considered alerts generated at prescribing and monitoring stages as we were interested in medication alerts for prescribers and pharmacists. Furthermore, these 2 stages hold the highest risk for ADEs.^29^^,^^30^ These alerts could warn for various risks, such as over- and underdosing, drug-drug interactions, contra-indications (including pregnancy and lactation), duplicate therapy, and drug intolerances and -allergies.

Regarding the use of AI-based methods, studies that did not report any performance measures or clinical outcomes were excluded. No restrictions were made to the type of performance measures or clinical outcomes used.

### Data extraction and synthesis

The titles and abstracts of the studies generated by the search strategy were collected and uploaded into Endnote© citation manager to remove duplicates. The first reviewer (J.G.) screened the titles and abstracts using ASReview version 1.0.^31^ ASReview is a tool designed to accelerate the screening of large numbers of literature references using active learning, a type of ML, with the main principle to achieve higher accuracy with fewer training data if the algorithm can choose the data from which it learns.^32^^,^^33^ The algorithm does not choose the included studies but merely presents the studies to the researchers in order of probability of relevance based on prior knowledge. It does not influence the search, but only uses the results of the search.

ASReview requires researchers to specify relevant and irrelevant papers related to a specific research question as prior knowledge, to train its algorithm. In this study, a total of 10 relevant and 10 irrelevant studies (as assessed by J.G.) were used to train the algorithm. Based on this prior knowledge the algorithm predicted a ranking of the relevance of all papers uploaded in the tool. Thereafter the screening of the title and abstract of the studies could start. van de Schoot et al reported that the number of relevant abstracts found after reading 10% of the abstracts ranges from 70% to 100%. Furthermore, 8% to 33% of all abstracts have to be screened to find 95% of the relevant studies.^30^ Following these findings, it was decided that after screening at least 10% of the abstracts and titles and 50 studies were consecutively identified as irrelevant based on the ranking of ASReview, the title and abstract screening process will be terminated (ie, stopping rule).

**Figure 1.**
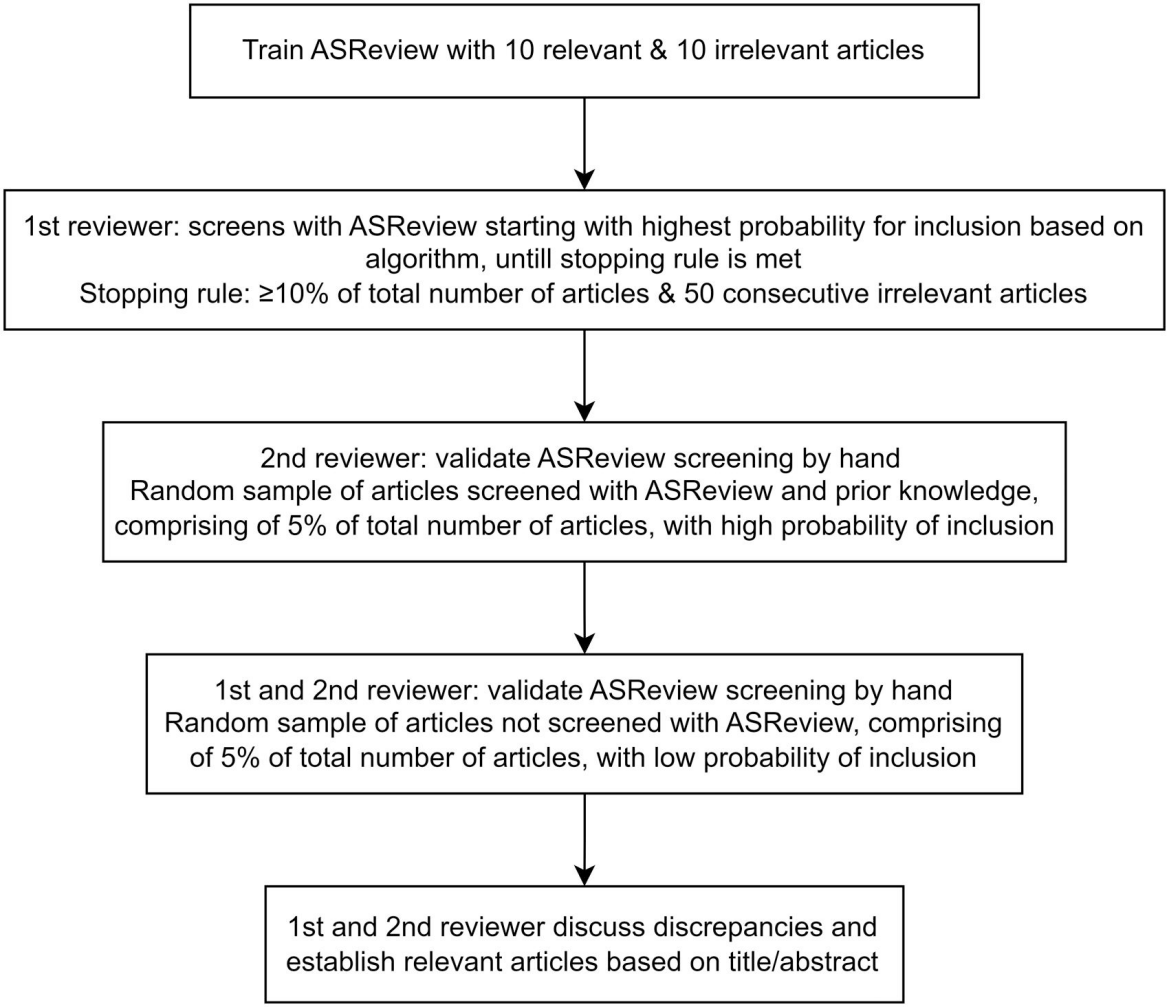
Process of title and abstract screening using ASReview software.

The results using ASReview ranking algorithm were verified by a second reviewer (R.M.) by manually screening a sample of the studies included through ASReview’s screening process, comprising 5% of the total number of studies and including the prior knowledge, to confirm that they would be included. Furthermore, the first and second reviewer (J.G. and J.M.) verified the results using ASReview by manually screening a random sample (comprising 5% of the total number of studies) of the studies excluded through ASReview’s screening process to confirm that they would be excluded. The process of the title and abstract screening is visualized in Figure 1.

At least 80% of the findings from the first and second reviewer had to be identical to validate the abstract screening and start the full text screening. If this was not the case, the first and second author had to screen another 10%. Subsequently, discrepancies in the findings were discussed to reach consensus and the inclusion criteria were reviewed and adjusted for clarification if needed.

Thereafter the full text screening and data extraction could start. The full-text review was done manually. Two reviewers (J.G., R.M.) screened 10% of the studies included in the full text screening to establish information to extract from the studies. The remaining studies for full-text screening were divided equally between these 2 reviewers. In case of doubt, the 2 reviewers tried to reach consensus. If this was not possible, third reviewer (P.v.d.B.) decided. All decisions were documented.

The information extracted from the included studies consisted of basic characteristics of the studies (title, author, affiliations, year of publication, journal, and country of origin), the study aims, study setting (eg, oncology, cardiology, intensive care, pediatrics, emergency), study methods, medication alerts characteristics (eg, drug-drug interactions, dosages, allergies alerts), AI-based methods used and their statistical performance, CDSS characteristics (eg, developer, targeted at physicians, pharmacist, or other healthcare professional), clinical outcomes studied and the findings, as well as most important conclusions. The extracted data were recorded in an Excel version 2307 (Microsoft, Redmond, WA, United States).

## Results

### Selection of included studies

**Figure 2.**
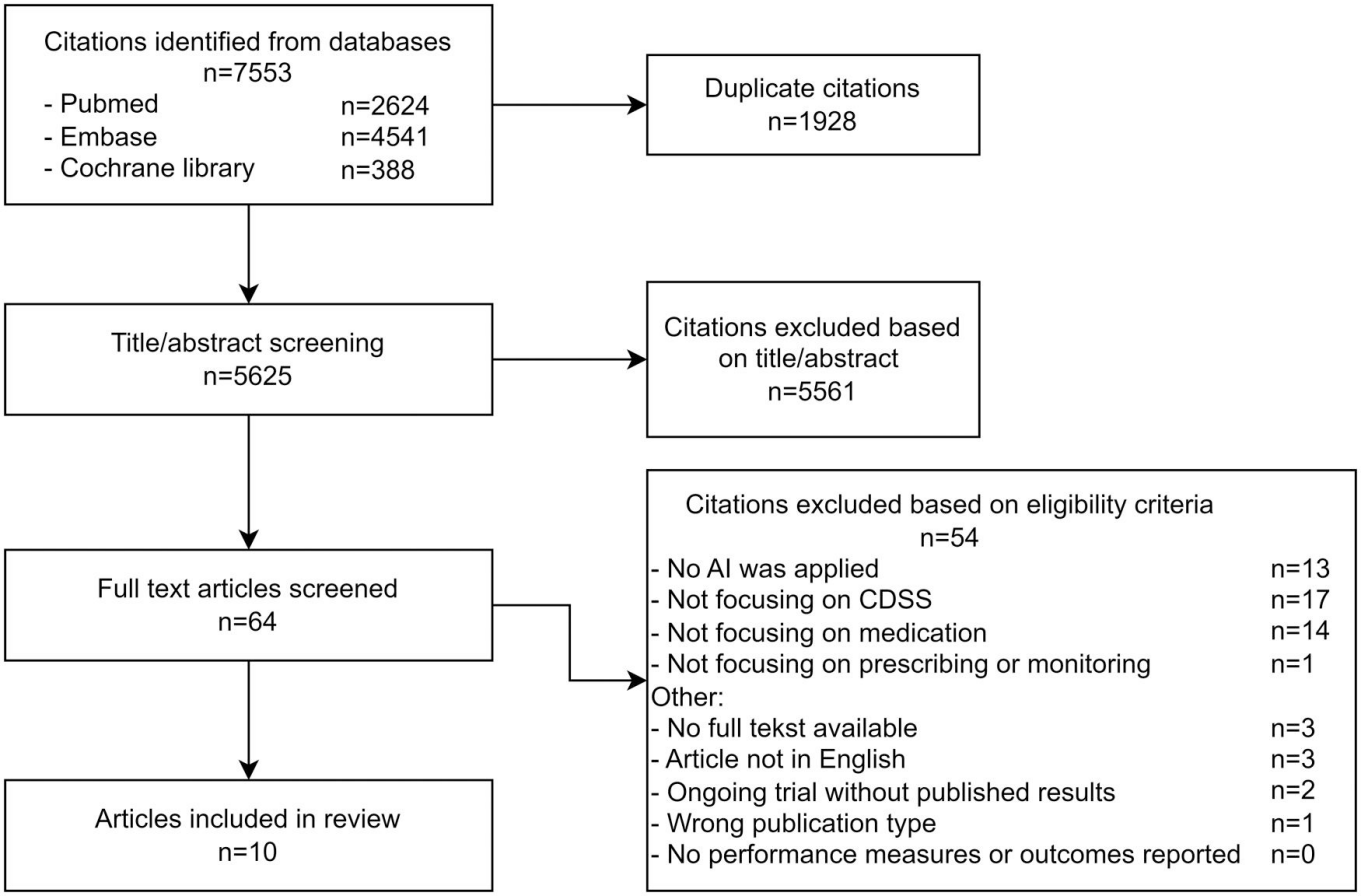
Preferred reporting items for systematic reviews and meta-analyses (PRISMA) flow diagram.

The flowchart of the selection process is shown in Figure 2. In total, 7553 citations were identified. After removal of 1928 duplicates, 5625 citations were left to be screened based on the title and abstract. After screening 10% of all citations in ASReview by the first reviewer, the stopping rule was reached since 126 subsequent irrelevant citations were found. The title and abstract screening eventually resulted in inclusion of 64 studies for full-text screening. Of these, 10 studies met the inclusion criteria and were included for data-extraction and analysis. No additional relevant studies were identified by checking references of relevant studies and forward citation in Web of Science and Scopus.

### Main characteristics of the included studies

In Table 1, a summary of the main characteristics of the 10 included studies is provided. The studies were published between 2013 and 2022. Nine studies (90%) were conducted in an academic or university hospital.^34–42^ Four studies (40%) stated the medical specialty studied.^34^^,^^38^^,^^40^^,^^42^ Segal et al included data from the internal medicine department^42^ and Lee et al from the pediatric department.^40^ Beaudoin et al focused on (inappropriate) antimicrobial prescriptions, in specific piperacillin-tazobactam prescriptions.^34^ Hogue et al included data from 7 different departments: obstetrics-gynecology and nursery, general pediatrics, surgery, oncology, specialized pediatrics, neonatal intensive care unit, and pediatric intensive care unit.^38^ The remaining 6 studies (60%) did not explicitly state the clinical domain or patient type studied.^35–37^^,^^39^^,^^41^^,^^43^ However, Kawazoe et al did mention including data from the 8 medicines most frequently causing alerts: ursodeoxycholic acid, carvedilol, sennoside, loxoprofen, brotizolam, nifedipine, famotidine, and pravastatin.^35^

**Table 1.** Main characteristics of the included studies.

Author	Year	Country	Study design	Setting	Specialty	Task	Data	
							Timeframe	Dataset
Segal et al^42^	2019	Israel	Prospective interventional study	An 1800-bed university medical center	Internal medicine	Evaluated an existing system	July 2016-April 2018	4533 admissions and 3160 patients with 78 017 medication orders
Schiff et al^36^	2017	United States	Retrospective, observational cohort study	2 university medical centers with a total of 1700 beds	Not stated	Evaluated an existing system	January 2009-December 2013	747 985 patients who had at least 1 visit during 2012-2013
Hogue et al^38^	2021	Canada	Prospective, international study	A 500 bed tertiary care mother-and-child university hospital at CHU	7 different departments: obstetrics-gynecology and nursery, general pediatrics, surgery, oncology, specialized pediatrics, NICU^a^, PICU^b^	Developed a model	Training data: 2005-2018 Testing data: April-August 2020	Training data: not stated Testing data: 12 624 medication orders and 2114 pharmacological profiles
Lee et al^40^	2022	Korea	Prospective interventional study	A university hospital	Pediatrics	Developed a model	Training data: January-November 2018 Testing data: December 2018	137 802 normal prescriptions and 1609 prescription errors
Kawazoe et al^35^	2013	Japan	Retrospective, observational study	A university hospital with >1200 beds	Not stated	Developed a model	January 2007-December 2011	Training data: 20 000 medication orders Testing data: 10 000 medication orders
Liu et al^41^	2022	United States	Prospective, interventional study	A medium-sized university medical center	Not stated	Evaluated an existing system	January-December 2019	3 481 634 medication alerts, 8270 providers, 178 298 patients Training data: 60%, validation data: 20%, testing data: 20%
Poly et al^37^	2020	Taiwan	Retrospective, observational study	A university medical center	Not stated	Developed a model	August 2018-May 2019	6453 prescriptions Training data: 60%, validation data: 20%, testing data: 20%
Corny et al^43^	2020	France	Retrospective observational study	A 592-bed hospital	Not stated	Developed a model	January 2017-August 2018	Training data: 94 720 hospitalizations, 61 611 patients Testing data: 412 patients with 3364 prescription orders
Balestra et al^39^	2021	United States	Prospective, observational study	A university medical center comprised of 3 hospitals with over 1600 beds	Not stated	Developed a model	10 July-24 July, 2017	181 407 individual orders submitted by 2708 providers Training data: 70%, validation data: 15%, testing data: 15%
Beaudoin et al^34^	2016	Canada	Prospective interventional study	A 677-bed secondary—and tertiary—care hospital located at 2 sites	Antimicrobial prescriptions	Evaluated a previously developed model	Training data: February-November 2012 Testing data: 18 November, 2013-20 December, 2013	Training data: 2092 patients, 2584 hospitalizations, and 4430 prescriptions Testing data: 350 patients, 421 hospitalizations, and 515 prescriptions

a NICU, neonatal intensive care unit.

b PICU, pediatric intensive care unit.

### Types of alerts optimized

Several types of medication alerts were generated by the CDSSs studied; over- and underdosing, drug-drug interactions, contra-indications (including pregnancy and lactation), duplicate therapy, and drug intolerances and -allergies. Except for the study by Kawazoe et al, where specifically dosing alerts were targeted, all others focused on optimizing the process related to the medication alerts. The study by Kawazoe et al specifically focused on optimizing dosing alerts by trying to identify appropriate dosing thresholds for the alerts.^35^

The approach for optimizing medication alerts varied between the studies. In 4 studies, the goal was to identify and prevent prescription errors.^36^^,^^38^^,^^40^^,^^42^ Liu et al, Poly et al, and Corny et al pursued similar objectives regarding refining the relevance of alerts; focusing on predicting physician’s responses^37^^,^^41^ or prioritizing prescription checks.^43^ Balestra et al developed a model based on past recommendations, focusing on designing a model for identifying medication orders requiring an intervention.^39^ Similarly, Beaudoin et al developed a model using past recommendations, evaluating a CDSSs consisting of a knowledge database linked to a model that extracts classification rules for alerts of inappropriate prescriptions.^34^

### CDSS used

In 2 studies a commercial system called Medaware^®^ (Raanana, Israel) was used.^37^^,^^42^ Corny et al tested the accuracy of Lumio Medication^®^, developed by Lumio Medical (Paris, France).^43^ Seven studies reported developing or validating a model themselves without explicitly naming the model (Table 2).^34^^,^^35^^,^^37–41^

**Table 2.** Type of alerts or process optimized and CDSS used.

Author	Type of alerts targeted	Prediction of	Name of CDSS	End-user	Type of model
Segal et al^42^	NA	Prescription errors	Medaware system	Physician	Model is used on top of regular CDSS
Schiff et al^36^	NA	Prescription errors	Medaware system	Physician	Model is used on top of regular CDSS
Hogue et al^38^	NA	Atypical drug orders and pharmacological profiles	Not stated	Pharmacist	Model is incorporated in CDSS
Lee et al^40^	NA	Prescription errors	Not stated	Physician	Hybrid system linking model to regular CDSS
Kawazoe et al^35^	Dosing alerts	NA	Not stated	Physician	Model is used on top of regular CDSS
Liu et al^41^	NA	User responses	Not stated	Not stated	Model is incorporated in CDSS
Poly et al^37^	NA	User responses	Not stated	Physician	Model is incorporated in CDSS
Corny et al^43^	NA	Prescription errors	Lumio Medication System	Pharmacist	Hybrid system linking model to regular CDSS
Balestra et al^39^	NA	Medication orders requiring interventions	Not stated	Pharmacist	Model is incorporated in CDSS
Beaudoin et al^34^	NA	Prescription errors	Not stated	Pharmacist	Model is used on top of regular CDSS

Abbreviation: NA = not applicable.

Five studies reported targeting the physicians^35–37^^,^^40^^,^^42^ and 4 targeting pharmacists.^34^^,^^38^^,^^39^^,^^43^ Liu et al aimed at filtering out alerts from the user’s view, without specifically mentioning who they were targeting as end-user.^41^

Four studies developed or studied a model to generate alerts that were normally not generated by the CDSS, which implies that the developed model is used on top of the regular CDSS.^34–36^^,^^42^ Four other studies developed a model aimed to be incorporated into the CDSS, by predicting user responses, by serving as a triage system or by filtering medication alerts generated by the regular CDSS.^37–39^^,^^41^ The 2 remaining studies developed a hybrid system linking an ML model to the regular CDSS.^40^^,^^43^

### AI-based methods used, their statistical performance, and outcomes

Nine studies reported using ML models (Table 3). Of these studies, 5 reported using supervised learning ML methods,^34^^,^^36^^,^^37^^,^^42^^,^^43^ Hogue et al reported using an unsupervised ML method,^38^ and 3 studies did not state whether they applied supervised or unsupervised ML.^35^^,^^39^^,^^41^ One study reported developing DL models based on supervised learning.^40^ None of the studies mentioned using NLP. The choice for these AI models and methods was not explicitly stated in the studies, but this generally depends on the specific requirements of the problem, the nature of the data, and the available computational resources. However, none of the included studies mentioned who chose the method of optimization nor was the field of expertise of the study team described.

**Table 3.** AI-based methods used, implementation, and validation.

Author	AI-based method used	**SL** ^a^ **or UL** ^b^	Implemented in practice	Validation
Segal et al^42^	ML	SL	Yes	Internal
Schiff et al^36^	ML	SL	No	Not performed
Hogue et al^38^	ML: GANomaly	UL	No	Internal
Lee et al^40^	DL: deep NN^c^ (eg, autoencoder)	SL	Yes	Internal
Kawazoe et al^35^	ML: bagging, CART^d^, RF^e^	Not stated	No	Internal
Liu et al^41^	ML: GBT^f^ (eg, LightGBM), NN^c^ (eg, autoencoder), RF^e^, SVM^g^	Not stated	No	Internal
Poly et al^37^	ML: GBT^f^, NN^c^ (eg, autoencoder), RF^e^, NB^h^	SL	No	Internal
Corny et al^43^	ML	SL	No	Internal
Balestra et al^39^	ML: GBT^f^ (eg, LightGBM)	Not stated	No	Internal
Beaudoin et al^34^	ML	SL	No	Internal

a SL, supervised learning.

b UL, unsupervised learning.

c NN, neural network.

d CART, classification and regression trees.

e RF, random forest.

f GBT, gradient boosted trees.

g SVM, support vector machine.

h NB, naïve Bayes.

Only 2 of the included studies reported implementing the developed AI-based model in daily practice (Table 3).^40^^,^^42^ Segal et al integrated Medaware into an existing EHR system in 1 university hospital in Israel. Initially, the system operated in a “silent mode” for several months.^41^ During this period analyses and monitoring were performed. Once the performance level was acceptable, the system switched to live mode in a single internal medicine department and the physicians started receiving alerts in the EHR system and could respond to them. The model by Lee et al was deployed in 1 university hospital in Korea, targeting pediatric outpatients and a selected number of medications.^40^

Seven studies reported outcomes, with the most often studied outcome (5 studies, 50%) being alert burden compared to the regular CDSS (Table 4).^35^^,^^40–43^ In these studies the alert burden was decreased by 14%-90%. Seven studies report statistical performance measures, in which a high variation is shown.^34^^,^^37–41^^,^^43^ The most often used performance measure was PPV broadly ranging from 9% to 100%. Lower PPV’s were reported in the studies of Hogue et al, Liu et al, and Balestra et al^38^^,^^39^^,^^41^ ranging from 9% to 49%, and higher PPV’s were reported in the studies of Lee et al, Poly et al, Corny et al, and Beaudoin et al ranging from 73% to 100%.^34^^,^^37^^,^^40^^,^^43^ Furthermore, the highest sensitivity and specificity of included studies were reported in the studies from Hogue et al (looking at the pharmacological profiles) and Poly et al ranging from 57% to 100%.^37^^,^^38^ Only 4 studies reported both statistical performance and outcomes.^34^^,^^40^^,^^41^^,^^43^

**Table 4.** Results, outcomes, and statistical performance measures reported.

Author	Results				Outcomes			Performance measures					
Segal et al^42^	Number of alerts as % of prescriptions: 37.10 for legacy CDS compared to 0.40 for Medaware. Additionally, the Medaware system performed surveillance on medication after prescribing, where the legacy CDS did not, comprising 60% of alerts.				% of alerts	Legacy CDS	Medaware	Not reported					

					Clinically relevant	16	85						
					Caused change in practice	5.30	43						
					Post prescribing surveillance^a^	0	60						
Schiff et al^36^	See clinical outcomes				Total of clinically valid alerts was 76.2% (96/126) of which 25% had less value, 18.8% had medium clinical value, and 56.2% had high clinical value.			Not reported					
Hogue et al^38^	See performance measures				Not reported			A: identifying atypical medication orders with AI					
								B: identifying pharmacological profiles with AI					
										A		B	
								PPV (%)		35		49	
								NPV (%)		96		93	
								Sensitivity (%)		26		75	
								Specificity (%)		97		82	
								AUROC		0.80		0.88	
								AUPR		0.25		0.60	
								F1		0.30		0.59	
Lee et al^40^	Number of alerts per month was reduced from 1613 (legacy CDSS) to 158 alerts (hybrid system)				Not reported			Prescription error detection					
								PPV (%)			73		
								Sensitivity (%)			81		
								F1			0.76		
Kawazoe et al^35^	Correlation coefficient = 0.80-0.95 at its highest for RF.^b^ The results indicate that when predicted thresholds for medication alerts are applied, in 4 drugs a reduction in alert volume will occur and in 4 drugs an increase will occur. In total, the predicted thresholds will reduce the alerts by half using the static threshold.				Not reported			Not reported					
Liu et al^41^	See clinical outcomes and performance measures				The proportion of alerts predicted as being non successful and thereby filtered out was 25 for LR, 14 for SVM, 31 for NN, 39 for RF, and 54 for GBT.			Predicting user responses to medication alerts					
									LR	SVM	NN	RF	GBT
								Probability threshold	0.02	0.04	0.01	0.15	0.06
								PPV^c^ (%)	12	10	13	15	19
								F1^c^	0.21	0.19	0.23	0.25	0.32
Poly et al^37^	See performance measures				Not reported			Predicting physicians response to CDSS alerts					
									NN	RF	NB	GBT	SVM
								PPV (%)	88	87	83	95	100
								NPV (%)	82	85	84	67	0.54
								Sensitivity (%)	87	88	87	79	57
								Specificity (%)	83	82	79	90	100
								Accuracy (%)	89	86	84	83	58
								F1	0.87	0.87	0.85	0.86	0.73
Corny et al^43^	The algorithm outperformed classic systems in its capacity to both detect patients with a medication error and to limit the number of false alerts. Of the remaining 26% prescription orders that required pharmacist intervention (false negatives) that were not intercepted by the algorithm, none were life-threatening.				Not reported			Identifying prescribing errors					
										CDSS		Lumio Medication	
								PPV (%)		54		74	
								Sensitivity (%)		69		74	
								AUROC (95%CI)		0.65 (0.61-0.69)		0.81 (0.78-0.84)	
								AUPR (95%CI)		0.56 (0.50-0.62)		0.75 (0.70-0.80)	
								F1		0.61		0.74	
Balestra et al^39^	See performance measures				Not reported			Identifying orders requiring intervention					
								PPV (%)			9		
								Sensitivity (%)			99		
								Specificity (%)			37		
								Accuracy (%)			41		
								AUROC			0.908		
								AUPR			0.439		
Beaudoin et al^34^	Reviewed prescriptions	B+L	B	L	Whereas the baseline system should be valued by its ability to identify a high proportion of inappropriate prescriptions (high recall) followed by its ability to trigger few false alerts (high precision), the learning module is valued by its ability to discover clinically relevant rules that complement those of the baseline system and to trigger actionable alerts.			Identifying confirmed inappropriate prescriptions					
	Prescriptions triggering alerts	270	240	105				(%(95% CI))		B+L		Baseline	Learning
	Rate of actionable alerts (%)	43	38	17				PPV		74 (68-79)		82 (77-87)	62 (52-71)
	The 17 prescriptions identified by the learning module included the 5 prescriptions that were missed by the knowledge base of the baseline system.	16	16	16				Sensitivity		96 (92-98)		94 (90-97)	31 (25-38)
								Accuracy		79 (74-83)		85 (81-89)	51 (46-56)

Abbreviations: PPV = positive predictive value (=precision), NPV = negative predictive value, Sensitivity = recall, AUROC = area under the receiver-operator curve, AUPR = area under the precision-recall, F1 = a metric in that balances precision (or PPV) and recall (or sensitivity), providing a value between 0 and 1, Correlation coefficient = coefficient between predicted doses and the actual doses, Probability threshold = a predefined value used in a classification model to decide the predicted class of an observation, with the model assigning the observation to one class if its predicted probability surpasses the threshold and to the other class otherwise. RF = random forest, LR = linear regression, SVM = support vector machine, NN = neural network, GBT = gradient boosted trees, NB = naïve Bayes, B = baseline, L = learning.

a Post prescribing surveillance = monitoring changing in the patient record to identify potential ADEs.

b RF performed better than the BAG and CART algorithm, all correlation coefficients are shown in figures in the original manuscript.

c Sensitivity value was set to ≥99%.

## Discussion

### Main findings

This scoping review provides a comprehensive overview of currently available evidence on the use of AI-based methods to optimize medication alerts generated by CDSS in the hospital setting. A decrease in alert burden was the most often studied outcome, varying from 14% to 90% in the included studies. Seven studies reported PPV as a performance measures, which broadly ranged from 9% to 100%. The highest PPV’s were reported in the studies of Lee et al, Poly et al, Corny et al, and Beaudoin et al ranging from 73% to 100%. These results indicate that AI-based methods have the potential to optimize medication alerts, but at the same time the results also show that there is substantial room for further improvement in application of these methods for this goal and reporting about such applications. Also, given the lack of external validation, the generalizability of the models and potential for implementation in hospital practice is limited.

To the best of our knowledge, this is the first review on the use of AI-based methods to optimize medication alerts generated by CDSS in the hospital setting. Several studies have been performed on AI in decision support systems in healthcare, but studies on medication alerts specifically are lacking. Furthermore, this review demonstrates that AI represents a novel approach to optimizing medication alerts. A recent scoping review of Ledger et al identified 6 types of interventions for optimization of medication alerts in hospitals; alert inactivation, alert severity reclassification, information provision, use of contextual information, threshold adjustment, and encounter suppression.^14^ AI is particularly well-suited for conducting these interventions, as is also shown by the studies included in this review.

The developed AI-based models have shown to decrease the alert burden and help identify more inappropriate or atypical prescriptions, compared to the regular CDSSs, subsequently leading to a decreased alert fatigue.^15^ In general, when more alerts are shown to physicians, they are less likely to intervene on these alerts.^44^ However, for AI-based medication alerts to be effective and safe, the models generating these alerts must have an optimal balance between sensitivity and specificity.^45^ The higher the specificity of the models, the less false alerts will be shown, leading to less alert fatigue. The higher the sensitivity, the better the models’ ability to produce alerts which warn about events with high probability of patient harm. None of the studies showed a sensitivity and specificity higher than 90%. This shows that optimizing medication alerts in the vast array of all prescriptions and medication alerts is a challenging task, also for AI-based methods.

Despite the promising results showing a decreased alert burden and high PPV values, in most studies the scope is limited as the study was conducted in a single hospital^36^^,^^37^^,^^39–43^ and/or with a focus on specific medication or departments.^40^^,^^42^ Furthermore, in only 2 studies, the optimized alerts or alert process were implemented in practice. Although not explicitly stated in the included studies, lack of alignment between the needs from hospital practice with AI-based efforts, and insufficient gains in terms of clinical outcomes may explain low uptake in hospital practice.^46^^,^^47^ Also, before an AI-based model can be adopted in hospital practice, extensive validation has to be performed, internally and externally. However, in most studies, the AI algorithm was developed using a limited dataset of only 1 hospital and none of the studies mention conducting external validation. Successful implementation may be achieved when more focus is given on the process of implementation in practice, rather than focusing solely on statistical performance measures.^43^^,^^45^ Also, the integration of the AI-based models into EHR systems to generate optimized medication alerts is challenging due to interoperability issues that often arise with such integrations.^46^^,^^48–50^

None of the included studies mentioned information about model development and validation. This limits transparency and replicability of the research and makes it difficult to assess the risk of bias and potential usefulness of the prediction model. This reflects a wider trend in reporting of clinical prediction model studies which has already been recognized for several years.^51^ Additionally, none of the included studies reported applying reporting guidelines such as Transparent reporting of a multivariable prediction model for individual prognosis or diagnosis (TRIPOD) Statement,^52^ which may explain why the relevant information about models development and validation was often missing. Adhering to the TRIPOD statement would ensure that enough information is provided for the reader to fully understand how the model was developed and validated. This transparency would support further research in the use of AI to improve CDSSs.

### Strengths, limitations, and future perspectives

Strengths of this scoping review include the comprehensive search in different electronic databases and the screening of the references of relevant studies together with forward citation. Furthermore, the title and abstract screening was validated by a second screener, to maintain consistency in the inclusion of studies. Additionally, the methodology and execution of this scoping review was structurally assessed since it was conducted in accordance with the JBI methodology for scoping review and the PRISMA-ScR.^26^^,^^27^

Limitations include the restriction to only include peer-reviewed studies. Non peer-reviewed studies may provide a more recent overview of the current state of AI-based methods for optimization of medication alerts generated by CDSS. On the other hand, novel methods for CDSSs in clinical settings must be assessed via rigorously peer-reviewed studies. Another limitation is the lack of critical appraisal of the included studies, but this is in accordance with the guidelines on scoping reviews we used.^26^^,^^27^ Also, given the high heterogeneity of studies included in terms of setting, methods and outcomes studies, a critical appraisal is of limited value. Furthermore, the utilization of ASReview may have resulted in missing relevant studies. Nonetheless, the inherent techniques of ASReview and the applied methodologies as described have mitigated this likelihood to a minimum. Additionally, these potentially missed studies were expected to be identified by forward citation. Finally, important contributions from Non-English speaking countries may have been missed.

Future studies on AI-based methods for optimization of medication alerts generated by CDSS in hospital settings should include larger datasets, to be able to extensively validate the models internally and externally. Prior to and during the development of CDSS using AI-based methods, it is essential to ensure sufficient support and collaboration with healthcare professionals, to facilitate trust, clinical value, and the implementation of the models in hospital practice. Moreover, such studies should use reporting guidelines, such as the upcoming ML focused TRIPOD (TRIPOD-ML) to enable critical appraisal of the results presented.^53^ Once the models are developed and validated, adequate attention must be given to their implementation into hospital practice.

## Conclusions

This scoping review provides an overview of the use of AI-based methods for optimizing medication alerts generated by CDSS in hospital setting. The then studies included show that AI has the capacity to adequately reduce alert burden and identify inappropriate prescriptions, but the datasets used were relatively small and the models lack formal validation. Most have not been implemented in hospital practice. Future studies should focus on validation and implementation of AI-based methods, and use reporting guidelines such as the TRIPOD Statement to report their work.

## Supplementary Material

ocae076_Supplementary_Data

## Data Availability

No new data were generated in support of this research. All relevant data are presented in the article.
