# Supplementary material for: The use of artificial intelligence to optimize medication alerts generated by clinical decision support systems: a scoping review
Source: J Am Med Inform Assoc. 2024 Apr 19;31(6):1411–22. doi: 10.1093/jamia/ocae076 (PMC11105146; doi:10.1093/jamia/ocae076)
Supplement: ocae076_Supplementary_Data [file ocae076_supplementary_data.docx]

## Appendix 1: search strategies

### Pubmed

### ("Clinical pharmacy information systems"[MeSH Terms] OR "Prescription Drug Monitoring Programs"[MeSH Terms] OR "Drug monitoring"[MeSH Terms] OR "Decision support techniques"[MeSH Terms] OR "Medical order entry systems"[MeSH Terms] OR "Prescription drug misuse"[MeSH Terms] OR "Drug interactions"[MeSH Terms] OR pharmacy information system*[Title/Abstract] OR "Medication Therapy Management"[Title/Abstract] OR medication manag*[tiab] OR drug manag*[tiab] OR Drug monitor*[Title/Abstract] OR decision support system*[Title/Abstract] OR Decision support technique*[Title/Abstract] OR Medical order entry system*[Title/Abstract] OR drug misus*[Title/Abstract] OR "Drug interaction*"[Title/Abstract] OR "Drug contraindication*"[Title/Abstract] OR "Medication Error*"[Title/Abstract] OR "Allerg*"[Title/Abstract] OR "Intoleran*"[Title/Abstract] OR "medication monitor*"[Title/Abstract] OR "medication surveillance*"[Title/Abstract] OR "Medication Therapy Management"[MeSH Terms] OR "decision support systems, clinical"[MeSH Terms] OR "contraindications, drug"[MeSH Terms] OR "Medication Errors"[MeSH Terms] OR "Allergy and Immunology"[MeSH Terms] OR drug safe*[Title/Abstract] OR prescription error*[tiab] OR “adverse drug events”[MeSH Terms] OR “Adverse drug events”[tiab] OR “drug related problems”[tiab] OR “Drug-related side effects and adverse reactions”[MeSH Terms]) NOT ("drug design"[Title/Abstract] OR "medication design"[Title/Abstract] OR "drug discovery"[Title/Abstract] OR "medication discovery"[Title/Abstract] OR "drug development"[Title/Abstract] OR "medication development"[Title/Abstract] OR "drug design"[MeSH Terms] OR "drug discovery"[MeSH Terms] OR "drug development"[MeSH Terms]) AND ("Pharmaceutical Services"[Mesh] OR "Pharmacists"[Mesh] OR "Pharmacy"[Mesh] OR pharma*[tiab] OR medication*[tiab] OR drug*[tiab]) AND ("artificial intelligence"[MeSH Terms] OR "artificial intelligen*"[Title/Abstract] OR "machine learning"[Title/Abstract] OR "deep learning"[Title/Abstract] OR "neural network*"[Title/Abstract] OR "unsupervised learning"[Title/Abstract] OR "supervised learning"[Title/Abstract] OR "expert system*"[Title/Abstract] OR "fuzzy logic"[Title/Abstract] OR "natural language process*"[Title/Abstract] OR "robotic*"[Title/Abstract] OR “data mining”[Title/Abstract] OR “text mining”[Title/Abstract] OR “machine intelligen*”[Title/Abstract] OR “prediction model*”[Title/Abstract] OR “reinforcement learning”[Title/Abstract] OR “recommender system*”[Title/Abstract] OR “statistical learning”[Title/Abstract] OR “computational intelligen*”[Title/Abstract] OR “computer reasoning”[Title/Abstract] OR “support vector machine*”[Title/Abstract] OR “multilayer perceptron*”[Title/Abstract] OR “random forest*”[Title/Abstract] OR “Bayesian network*”[Title/Abstract] OR “nearest neighbor*”[Title/Abstract] OR “elastic net”[Title/Abstract] OR “naive bayes”[Title/Abstract])

### Embase

(('medical information system'/exp OR 'drug monitoring'/exp OR 'decision support system'/exp OR 'physician order entry system'/exp OR 'prescription drug misuse'/exp OR 'drug interaction'/exp OR 'medication therapy management'/exp OR 'clinical decision support system'/exp OR 'drug contraindication'/exp OR 'immunology'/exp OR ‘adverse drug reaction’/exp) OR (‘pharmacy information system*’ OR ‘Medication Therapy Management’ OR ‘medication manag*’ OR ‘drug manag*’ OR ‘Drug monitor*’ OR ‘decision support system*’ OR ‘Decision support technique*’ OR ‘Medical order entry system*’ OR ‘drug misus*’ OR ‘Drug interaction*’ OR ‘Drug contraindication*’ OR ‘Medication Error*’ OR Allerg* OR Intoleran* OR ‘medication monitor*’ OR ‘medication surveillance*’ OR ‘drug safe*’ OR ‘prescription error*’ OR ‘adverse drug event*’ OR ‘drug related problem*’):ab,ti,kw) NOT ((‘drug design’/exp OR ‘drug development’/exp) OR (‘drug design’ OR ‘medication design’ OR ‘drug discovery’ OR ‘medication discovery’ OR ‘drug development’ OR ‘medication development’):ab,ti,kw) AND ('pharmacy (shop)'/exp OR 'pharmacist'/exp OR (pharma* OR medication* OR drug*):ab,ti,kw) AND ('artificial intelligence'/exp OR 'artificial neural network'/de OR 'back propagation'/de OR 'data mining'/de OR 'random forest'/de OR 'semi supervised machine learning'/de OR 'supervised machine learning'/de OR 'support vector machine'/de OR (‘artificial intelligen*’ OR ‘machine learning’ OR ‘deep learning’ OR ‘neural network*’ OR ‘unsupervised learning’ OR ‘supervised learning’ OR ‘expert system*’ OR ‘fuzzy logic’ OR ‘natural language process*’ OR robotic* OR ‘data mining’ OR ‘text mining’ OR ‘machine intelligen*’ OR ‘prediction model*’ OR ‘reinforcement learning’ OR ‘recommender system*’ OR ‘statistical learning’ OR ‘computational intelligen*’ OR ‘computer reasoning’ OR ‘support vector machine*’ OR ‘multilayer perceptron*’ OR ‘random forest*’ OR ‘Bayesian network*’ OR ‘nearest neighbor*’ OR ‘elastic net’ OR ‘naive bayes’):ab,ti,kw) NOT 'conference abstract'/it

### Cochrane library

(“pharmacy information system” OR “pharmacy information systems” OR "Medication Therapy Management" OR “medication management” OR “drug management” OR “Drug monitor” OR “drug monitors” OR “drug monitoring” OR “decision support system” OR “decision support systems” OR “Decision support technique” OR “Decision support techniques” OR “Medical order entry system” OR “Medical order entry systems” OR “drug misuse” OR "Drug interaction” OR "Drug interactions” OR "Drug contraindication” OR "Drug contraindications” OR "Medication Error” OR "Medication Errors” OR Allerg* OR Intoleran* OR "medication monitor" OR "medication monitors" OR "medication monitoring" OR "medication surveillance*" OR "medication surveillances” OR “drug safety” OR “prescription error” OR “prescription errors” OR “adverse drug event*” OR “drug related problem*”) AND (pharma* OR medication* OR drug*) AND ("artificial intelligence” OR "machine learning" OR "deep learning" OR "neural network” OR “neural networks” OR "unsupervised learning" OR "supervised learning" OR "expert system” OR “expert systems” OR "fuzzy logic" OR "natural language process” OR "natural language processing” OR robotic* OR “data mining” OR “text mining” OR “machine intelligence” OR “prediction model” OR “prediction models” OR “reinforcement learning” OR “recommender system” OR “recommender systems” OR “statistical learning” OR “computational intelligence” OR “computer reasoning” OR “support vector machine” OR “support vector machines” OR “multilayer perceptron” OR “multilayer perceptrons” OR “random forest” OR “random forests” OR “Bayesian network” OR “Bayesian networks” OR “nearest neighbor” OR “nearest neighbors” OR “elastic net” OR “naive bayes”)
